# Novel autosomal dominant mutation in loricrin presenting as prominent ichthyosis

**DOI:** 10.1111/bjd.13895

**Published:** 2015-08-22

**Authors:** E. Pohler, F. Cunningham, A. Sandilands, C. Cole, S. Digby, J.R. McMillan, S. Aristodemou, J.A. McGrath, F.J.D. Smith, W.H.I. McLean, C.S. Munro, M. Zamiri

**Affiliations:** ^1^ Centre for Dermatology and Genetic MedicineColleges of Life Sciences and Medicine, Dentistry and NursingUniversity of DundeeU.K.; ^2^ Department of DermatologyUniversity Hospital CrosshouseKilmarnock RoadKilmarnockKA2 0BEU.K.; ^3^ Division of Computational BiologyCollege of Life SciencesUniversity of DundeeU.K.; ^4^ Department of PathologySouthern General HospitalGlasgowU.K.; ^5^ EB LaboratoryViapath LLPSt Thomas' HospitalLondon GroupKing's College London (Guy's Campus)LondonU.K.; ^6^ Department of Cell PathologySt John's Institute of DermatologySt. Thomas' HospitalLondonU.K.; ^7^ Alan Lyell Centre for DermatologySouthern General HospitalGlasgowU.K.

dear editor, Loricrin keratoderma (syn. Camisa syndrome, OMIM 604117) is a rare autosomal dominant genodermatosis characterized by palmoplantar keratoderma and ichthyosis.^1^, ^2^ It is caused by mutations in loricrin, a small basic protein synthesized in the upper granular layer, which becomes a major constituent of the cornified cell envelope.^3^ Seven distinct mutations in loricrin have been reported in 15 unrelated pedigrees to date.^4^, ^5^, ^6^ We report a multi‐generation family with prominent ichthyosis and palmoplantar involvement due to a novel mutation in loricrin.

The proband was a 14‐year‐old boy who presented with generalized dryness and scaling affecting his trunk and all four limbs, previously thought to be ichthyosis vulgaris, which was reported from early childhood (Fig. 1a). Clinical examination revealed widespread, prominent ichthyosis and mild diffuse transgredient hyperkeratosis of palms and soles. There was no evidence of atopic dermatitis or keratosis pilaris. His mother and eight other family members were similarly affected (Fig. 1b). Subsequently, a further child was born with a collodion membrane followed by generalized ichthyosis.

**Figure 1:**
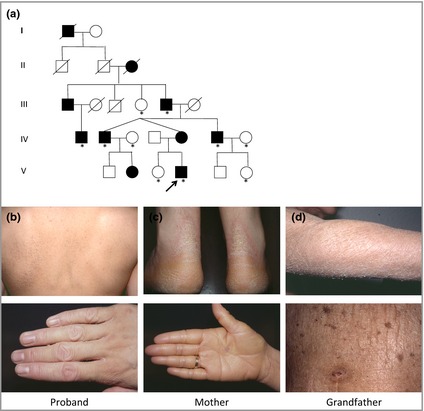
(a) Pedigree of family with loricrin keratoderma. Filled symbols represent affected individuals. Whole exome sequencing was performed on one affected family member. *marks individuals who were screened for loricrin mutation by Sanger sequencing. (b–d) Clinical pictures of (b) the proband: ichthyosis of upper back with brawny appearance and knuckle pads; (c) proband's mother: transgredient hyperkeratosis of palms and soles; (d) proband's grandfather: widespread ichthyosis of forearm and trunk.

Biopsies of affected skin were processed for light and electron microscopy by standard methods (upper back of the proband's mother) or for electron microscopy only (affected acral skin from the proband's grandfather).^7^ Following informed consent, genomic DNA was extracted from blood or saliva samples from 10 affected and unaffected family members (Fig. 1a). A whole exome sequencing approach was taken to analyse the proband's DNA (Methods S1; see Supporting Information).

Light microscopy of skin from the proband's mother showed mild hyperkeratosis, a normal granular layer and no significant parakeratosis (Fig. 2a). Electron microscopy of (i) affected acral skin demonstrated mild intracellular oedema, abundant keratohyaline granules in upper layers, with desmosomes and keratin filaments appearing intact and of (ii) affected upper back skin, vacuolar changes and disruption of suprabasal keratinocytes (Fig. 3). Whole exome sequencing directed at relevant epidermal genes revealed a novel heterozygous duplication mutation in the loricrin gene in exon 2 (designated c.806dupG), with an insertion of a single base pair resulting in a frameshift leading to a delayed termination codon and elongation of the protein by 22 amino acids (Fig. 2d). The mutation was confirmed by Sanger sequencing (Methods S2; see Supporting Information) and was present in affected individuals but was not in unaffected family members (Figs 1a, 2b,c). This mutation is not on the dbSNP database or NHLBI Exome Variant Server (http://evs.gs.washington.edu/EVS/).

**Figure 2:**
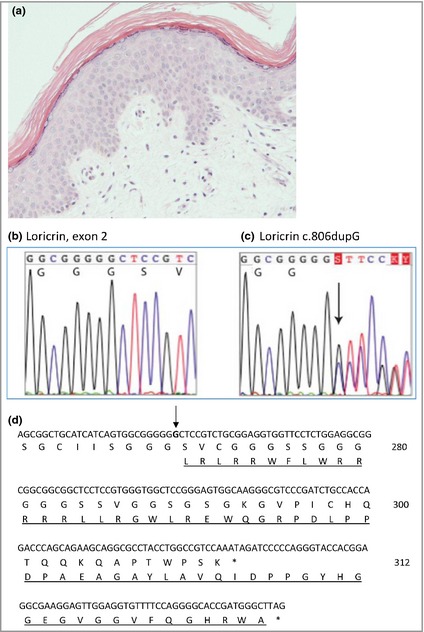
(a) A biopsy from the proband's mother's skin showing mild hyperkeratosis, a normal granular layer and no significant parakeratosis (original magnification × 20). (b–d) Mutation analysis. (b) Normal loricrin sequence in exon 2, showing nucleotides 799–813 (codons 267–271). (c) The equivalent region as in (b) from the proband showing the heterozygous mutation c.806dupG, leading to a delayed termination codon and a mutant protein 22 amino acids longer than wild‐type. (d) Nucleotide sequence at the 5′ end of loricrin. The inserted G nucleotide is in bold and indicated with an arrow. The predicted amino acid sequences of the wild‐type and mutant (underlined) alleles are shown.

**Figure 3:**
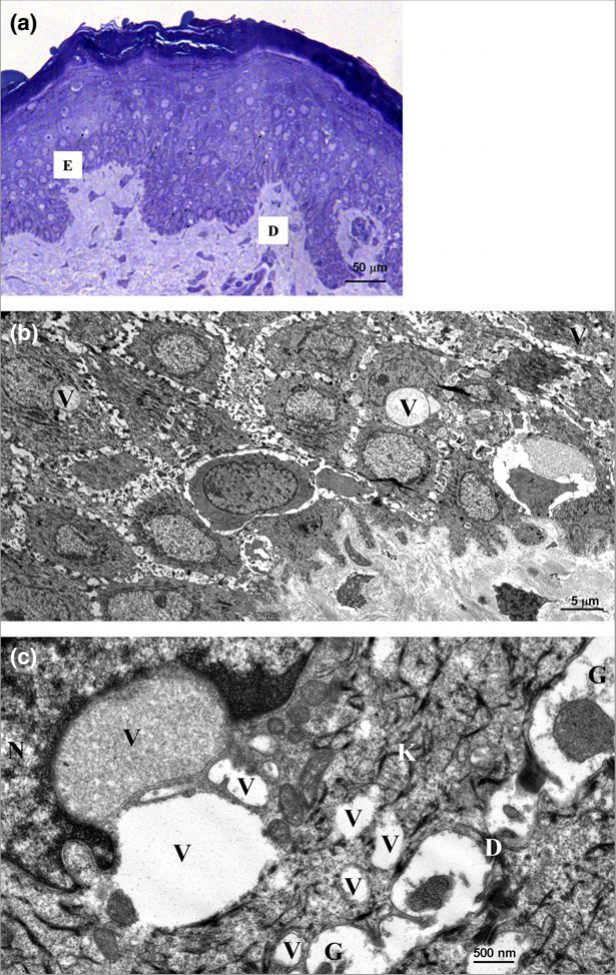
Semithin sections and ultrastructural images. In semithin sections (a) there was epidermal acanthosis and compact hyperkeratosis with a pattern of diffuse vacuolar change. These vacuoles (small arrows) were predominantly observed in the suprabasal keratinocyte layers. Suprabasal layer keratinocytes also showed focal cytolytic changes and some cells appeared necrotic. (b) Ultrastructurally there was disruption to the normal keratin filament network within suprabasal keratinocytes concurrent with the presence of peri‐nuclear, membrane‐bound vacuoles (V), some containing granular material. At higher magnification (c), keratin filament networks (K) were disturbed or showed some clumping particularly around the nucleus (N). Desmosome connections between keratinocytes mostly appeared normal, although there were small detached desmosomes (D) with disrupted keratin filament (K) association in some areas together with a slight widening of intercellular spaces between adjacent keratinocytes and some granular debris (G) present within the intercellular spaces. Scale bars 50 μm (a), 5 μm (b) and 500 nm (c).

Six different heterozygous insertion mutations in loricrin in 14 unrelated pedigrees have previously been reported and one heterozygous deletion in a further pedigree^2^, ^4^, ^5^, ^6^, ^8^, ^9^, ^11^, ^12^, ^13^ (see Supporting Information; Table S1). All six insertion mutations are single base‐pair insertions leading to delayed termination codons with the most frequent mutation 730insG being present in eight of the 14 published families.^2^, ^4^, ^8^, ^9^, ^11^, ^12^, ^13^ This region of the loricrin gene is thought to be a mutation hotspot because of the presence of six consecutive guanine nucleotides.^12^All single‐base‐pair insertion and deletion mutations lead to a frameshift and delayed termination, thus elongating the protein by 22 amino acids and changing the Gly‐Lys‐rich domain into an Arg‐Leu‐rich terminal domain,^2^ except for two pedigrees of Brazilian origin, where the new protein is 25 amino acids longer than wild‐type protein.^5^ The mechanism of action of these mutations is thought to relate to preferential localization of mutant loricrin in the nucleus due to the formation of nuclear localization sequences within the arginine‐rich mutant loricrin.^14^ It has been suggested that the abnormal nuclear protein may disrupt the apoptotic process in terminal differentiation of keratinocytes in mouse models, thus supporting the hypothesis that the phenotype of loricrin keratoderma is caused by the synthesis of mutant loricrin rather than by the lack of wild‐type loricrin.^14^, ^15^ No clear genotype‐phenotype associations for pedigrees with specific mutations have currently been identified.^4^, ^5^, ^13^

The phenotype of loricrin keratoderma is heterogeneous. The common clinical features in both the 15 previously reported pedigrees and our new pedigree are palmoplantar keratoderma, usually of a honeycomb pattern, and generalized ichthyosis. Other features, including knuckle pads, pseudoainhum/hyperconstricting bands with autoamputation of digits and collodion babies, are variably reported. Previously, Gedicke *et al*.^13^ have suggested that the term ‘mutilating keratoderma with ichthyosis’ is not entirely suitable, due to the variation in phenotypes previously reported. Similarly, given the prominence of generalized ichthyosis with lesser palmoplantar involvement in this pedigree, and the presence of generalized ichthyosis in all previously reported pedigrees, we suggest that the condition could be described as loricrin ichthyosis rather than loricrin keratoderma.

## Supporting information


**Methods S1.** Whole exome sequencing.

**Methods S2.** Confirmation of mutation in exon 2 by Sanger sequencing

**Table S1.** Clinical features of families with loricrin keratoderma.
