# Supplementary material for: Novel autosomal dominant mutation in loricrin presenting as prominent ichthyosis
Source: Br J Dermatol. 2015 Aug 22;173(5):1291–4. doi: 10.1111/bjd.13895 (PMC4832349; doi:10.1111/bjd.13895)
Supplement: Supplementary file 1 — Methods S1. Whole exome sequencing. [file BJD-173-1291-s001.docx]

**Supplementary Material**

**Methods**

Genomic DNA was extracted from peripheral blood leukocytes using standard procedures or from saliva and extracted according to the manufacturer’s protocol. Samples were obtained with informed consent and ethical approval by a Western Institutional Review Board that complies with principles of the Helsinki Accord.

Whole exome sequencing – WES was performed with Agilent SureSelect v4 capture kit and sequenced on an Illumina HiSeq 2000 (GenePool, Edinburgh). The resulting 88.7M 100 bp PE sequencing reads were aligned to the human genome (Ensembl release 68) with Bowtie2 (v2.02) (98.8% aligned)^1.^ Read duplicates were removed with Picard Tools (v1.79) ([http://picard.sourceforge.net](http://picard.sourceforge.net/)) and variants were called using the UnifiedGenotyper in GATK-lite (v2.2-8) following the GATK ‘best practices’^2.^ The 69,732 called variants were annotated with the Variant Effect Predictor v72 ^3^ and any variants with the following consequences were filtered out: downstream_gene_variant, upstream_gene_variant, synonymous_variant, intergenic_variant and intron_variant. Filtered data were put in a MySQL database allowing querying via a Django interface.

References

1.Langmead, Ben, and Steven L. Salzberg. ‘Fast Gapped-Read Alignment with Bowtie 2’. *Nature Methods* 9, no. 4 (April 2012): 357–59. doi:10.1038/nmeth.1923.

2. McKenna A, Hanna M, Banks E*, et al.* (2010) The Genome Analysis Toolkit: a MapReduce framework for analyzing next-generation DNA sequencing data. *Genome Research* 20:1297-303.

3. McLaren W, Pritchard B, Rios D*, et al.* (2010) Deriving the consequences of genomic variants with the Ensembl API and SNP Effect Predictor. *Bioinformatics* 26:2069-70.
