# Supplementary material for: Novel autosomal dominant mutation in loricrin presenting as prominent ichthyosis
Source: Br J Dermatol. 2015 Aug 22;173(5):1291–4. doi: 10.1111/bjd.13895 (PMC4832349; doi:10.1111/bjd.13895)
Supplement: Supplementary file 2 — Methods S2. Confirmation of mutation in exon 2 by Sanger sequencing [file BJD-173-1291-s002.docx]

**Method S2**

Confirmation of mutation in exon 2 by Sanger sequencing

A 1367 bp fragment spanning exon 2 of loricrin was amplified using primers LorEx2_F1 5^’^ GATGTTGCCTATGGATGCAGC 3’ and LorEx2_R1 5’ GTAAGTGTGAATGAGCGAATGC 3’ using a GC-Rich PCR System (Roche, Germany) and the following cycling conditions: 94°C 5min; 35 cycles of 94°C 30s, 56°C 30 sec, 72°C 1min; 72°C 5min. PCR products were purified using QiaQuick PCR spin columns (Qiagen House, Crawley, UK) and sequenced on an ABI 3700 Automated DNA sequencing machine (Foster City, CA) according to the manufacturer’s instructions, using additional primers LorEx2_F2 5’ GGTGGCTGCGGCTTCTTC 3’ and LorEx2 F3 5’ GTCTCCTCGCAGCAGGTC 3’.
