# Supplementary material for: Novel autosomal dominant mutation in loricrin presenting as prominent ichthyosis
Source: Br J Dermatol. 2015 Aug 22;173(5):1291–4. doi: 10.1111/bjd.13895 (PMC4832349; doi:10.1111/bjd.13895)
Supplement: Supplementary file 3 — Table S1. Clinical features of families with loricrin keratoderma. [file BJD-173-1291-s003.docx]

| **Family** | **Position of base pair insertion** | **Ichthyosis** | **Honeycomb-like PPK** | **PPK** | **Collodion baby** | **Special** **Skin abnormalities** | **Reference** |
| --- | --- | --- | --- | --- | --- | --- | --- |
| 1 | 730 | + | + | + | - | - | 2 |
| 2 | 730 | + | + | + | + | - | 8 |
| 3 | 709 | + | + | + | - | Erythematous  plaques | 16 |
| 4 | 662 | + | + | + | - | Hyperkeratosis over knuckles | 17 |
| 5 | 730 | + | + | + | - | - | 11 |
| 6 | 730 | + | + | + | + | - | 8 |
| 7 | 730 | + | + | + | - | - | 9 |
| 8 | 730 | + | + | + | + | Ichythosiform erythroderma | 15 |
| 9 | 730 | + | + | + | - | Increased knuckle markings | 12 |
| 10 | 578 | + | - | + | - | Hyperkeratosis  Dorsa hands | 18 |
| 11 | 730 | + | + | + | + | Erythematous plaques | 4 |
| 12 | 646 | + |  | + | + | Yellow and more severe hyperkeratosis in  body folds | 5 |
| 13 | 798 | + |  | + | + | - | 5 |
| 14 |  | + |  | + | - | - | 5 |
| 15 | Micro deletion 660 | + | + | + | + | - | 6 |
| 16 | 806 | + | + | + | + | Transgredient hyperkeratosis of palms and soles | Present study |
